# Prognostic impact of adjacent organ invasion on cancer-specific mortality in non-metastatic renal cell carcinoma

**DOI:** 10.1186/s12957-026-04511-3

**Published:** 2026-07-20

**Authors:** Maximilian Filzmayer, Leonardo Quarta, Michele Petix, Filippo Orlandi, Jordan A. Goyal, Alberto Briganti, Luca Carmignani, Salvatore Micali, Shahrokh F. Shariat, Matthias J. Müller, Clara Humke, Miriam I. Traumann, Fred Saad, Felix K.-H. Chun, Pierre I. Karakiewicz

**Affiliations:** 1 Cancer Prognostics and Health Outcomes Unit, Division of Urology, University of Montreal Health Center, Montreal, Canada; 2 Department of Urology, Goethe University Frankfurt, University Hospital, Frankfurt am Main, Germany; 3 Vita-Salute San Raffaele University, Milan, Italy; 4 Division of Experimental Oncology/Unit of Urology, URI, Urological Research Institute, IRCCS San Raffaele Scientific Institute, Milan, Italy; 5 Università degli Studi di Milano, Milan, Italy; 6 Department of Urology, IRCCS Policlinico San Donato, Milan, Italy; 7 Department of Urology, AOU di Modena, University of Modena and Reggio Emilia, Modena, Italy; 8 Department of Urology, Comprehensive Cancer Center, Medical University of Vienna, Vienna, Austria; 9 Department of Urology, Weill Cornell Medical College, New York City, USA; 10 Department of Urology, University of Texas Southwestern Medical Center, Dallas, USA; 11 Hourani Center of Applied Scientific Research, Al-Ahliyya Amman University, Amman, Jordan

**Keywords:** Renal cell carcinoma, Nephrectomy, Pathological T4 stage, Adjacent organ invasion, Cancer-specific mortality, Adjuvant therapy, SEER

## Abstract

**Purpose:**

The increase in cancer-specific mortality (CSM) associated with pathologically confirmed adjacent organ invasion (pT4) in non-metastatic (M0) renal cell carcinoma (RCC), relative to limited local invasion (pT3) or no local invasion (pT2) with otherwise same patient and tumor characteristics, is unknown. We addressed this knowledge gap.

**Methods:**

Within the Surveillance, Epidemiology, and End Results database (2004–2022), M0 RCC patients treated with radical nephrectomy were identified. Propensity score matching (PSM) was applied, and multivariable competing-risks regression (CRR) models were fitted. Additional sensitivity analyses were performed to evaluate the robustness of the primary findings.

**Results:**

Of 27,859 patients, 666 (2.4%) were classified as pT4, 15,343 (55.1%) as pT3, and 11,850 (42.5%) as pT2. After PSM, CSM at 120 months was 63.0% in pT4 versus 43.2% in pT3 and 26.0% in pT2. In multivariable CRR models, pT4 was associated with 1.84-fold higher CSM compared with pT3 (*p* < 0.001) and 3.31-fold higher CSM compared with pT2 (*p* < 0.001). In sensitivity analyses restricted to patients with clear-cell RCC, patients diagnosed before approval of adjuvant systemic therapy, and patients without lymph node invasion, corresponding effect estimates ranged from 1.79 to 1.93 for the comparison with pT3 and from 2.96 to 3.47 for the comparison with pT2 (all *p* < 0.001).

**Conclusion:**

In M0 RCC, the presence of adjacent organ invasion (pT4) doubles the rate of CSM relative to pT3 and triples the rate of CSM relative to pT2 when all other tumor and patient characteristics are held constant. These findings quantify the very aggressive nature of pT4 independent of other tumor and patient characteristics and thereby validate contemporary T staging. Accordingly, more frequent follow-up and prioritization of pT4 patients for discussions regarding adjuvant systemic therapy may be warranted compared with their pT3 and pT2 counterparts.

**Supplementary Information:**

The online version contains supplementary material available at 10.1186/s12957-026-04511-3.

## Introduction

Pathological T4 (pT4) stage in renal cell carcinoma (RCC) is defined by tumor invasion beyond Gerota’s fascia and into adjacent organs, and represents the most extensive form of local invasion within the TNM classification [1]. Although adjacent organ invasion is occasionally suspected preoperatively in large renal masses, many clinically staged T4 (cT4) tumors are downstaged to pT3 or pT2 at final pathology [2–9]. True pT4 is rare in non-metastatic (M0) patients [8–16].

Stage pT4 has consistently been associated with higher cancer-specific mortality (CSM) [8–15]. However, existing evidence largely derives from TNM validation studies comparing pT4 tumors to low-stage tumors and within heterogeneous cohorts including metastatic patients. Consequently, it is unknown to what extent presence of pT4 increases the risk of CSM relative to limited local invasion (pT3) when all other characteristics are held constant. The same uncertainty exists in the comparison with organ-confined tumors (pT2) in patients who otherwise exhibit all the same characteristics as their pT4 counterparts. To address this knowledge gap, we conducted a population-based analysis using the Surveillance, Epidemiology, and End Results (SEER) database (2004–2022) [17].

## Materials and methods

### Study population and outcomes of interest

We identified adult (≥ 18 years) patients with histologically confirmed pT2–4 RCC (International Classification of Diseases for Oncology Third Edition site code C64.9) treated with radical nephrectomy (*n* = 42,790) [17]. Patients with evidence of distant metastases at diagnosis, including non-contiguous adrenal gland involvement, were excluded (*n* = 5,724). Additional exclusion criteria consisted of missing information regarding patient and tumor characteristics (*n* = 8,448) or follow-up (*n* = 759). Consequently, a complete-case analysis approach was applied. The outcome of interest was CSM, defined as death attributable to RCC as recorded within the SEER database. CSM rates were estimated at 120 months from the date of nephrectomy.

### Statistical analyses

The analyses consisted of two separate comparisons: pT4 versus pT3 and pT4 versus pT2. In both comparisons, one-to-one propensity score matching (PSM) using the nearest neighbor method without replacement was applied, first between pT4 and pT3 and subsequently between pT4 and pT2. PSM relied on year of diagnosis (continuous), age (continuous), sex (male vs. female), race/ethnicity (Caucasian vs. non-Caucasian), marital status (married vs. unmarried), income (low [annual income below the cohort median of 80,000 USD] vs. high), area of residence (urban vs. rural), tumor size (continuous), histological subtype (clear-cell vs. papillary vs. chromophobe vs. variant histology), tumor grade (G1–2 vs. G3–4 vs. Gx), and lymph node invasion (pN+ vs. pN0 vs. pNx). After PSM, cumulative incidence plots depicted differences in CSM rates and multivariable Fine-Gray competing-risks regression (CRR) models were fitted to test the effect of pT4 on CSM [18]. Other-cause mortality (OCM) was defined as death from non-RCC-related causes and was treated as a competing risk event. Three additional analyses were performed to evaluate the robustness of the primary findings. First, all analyses were repeated in patients with clear-cell RCC only. Second, all analyses were repeated in patients diagnosed between 2004 and 2017, before the widespread adoption of adjuvant systemic therapy [19–22]. Third, all analyses were repeated after exclusion of patients with lymph node invasion.

Data extraction was performed using SEER*Stat software version 8.4.5 (RRID: SCR_006902; National Cancer Institute, Bethesda, USA). Statistical analyses and graphics were generated using R software version 4.5.2 (RRID: SCR_001905; R Foundation for Statistical Computing, Vienna, Austria). All tests were two-sided with significance set at *p* < 0.05 and Bonferroni correction was applied for multiple comparisons.

### Ethical approval and consent to participate

The study was conducted in accordance with the Declaration of Helsinki. Ethical approval for this study and requirement for informed consent was waived by the Institutional Review Board (Comité d’éthique à la recherche du Centre hospitalier de l’Université de Montréal) due to the retrospective nature of the study and the use of anonymized data from the SEER database.

## Results

### Descriptive characteristics

Within the SEER database (2004–2022), 27,859 patients undergoing radical nephrectomy for M0 RCC were identified (Table 1). Of these, 666 patients (2.4%) harbored pT4, while 15,343 (55.1%) were classified as pT3 and 11,850 (42.5%) as pT2.

**Table 1:** Descriptive characteristics of pT2–4 renal cell carcinoma patients treated with radical nephrectomy within the SEER database (2004–2022), stratified according to pT4 versus pT3 versus pT2, before propensity score matching

Characteristics	Overall *n* = 27,859	pT4 *n* = 666 (2.4%)	pT3 *n* = 15,343 (55.1%)	*p*-value ^a^	pT2 *n* = 11,850 (42.5%)	*p*-value ^a^
Year of diagnosis, median (IQR)	2015 (2010, 2019)	2012 (2008, 2018)	2014 (2010, 2020)	< 0.001	2015 (2009, 2017)	< 0.001
Age (in years), median (IQR)	63 (54, 71)	62 (55, 72)	64 (56, 72)	0.002	61 (52, 69)	< 0.001
Male sex, n (%)	18,970 (68.1%)	431 (64.7%)	10,705 (69.8%)	0.005	7,834 (66.1%)	0.5
Non-Caucasian race/ethnicity, n (%)	9,295 (33.4%)	234 (35.1%)	4,878 (31.8%)	0.1	4,183 (35.3%)	> 0.9
Married, n (%)	18,190 (65.3%)	412 (61.9%)	10,101 (65.8%)	0.035	7,677 (64.8%)	0.1
Low income ^b^, n (%)	14,198 (51.0%)	356 (53.5%)	7,743 (50.5%)	0.1	6,099 (51.5%)	0.3
Rural residence, n (%)	3,782 (13.6%)	90 (13.5%)	2,258 (14.7%)	0.4	1,434 (12.1%)	0.3
Tumor size (in cm), median (IQR)	8.5 (7.2, 10.5)	10.5 (8.0, 13.2)	8.0 (6.0, 10.0)	< 0.001	9.0 (8.0, 11.0)	< 0.001
Histological subtype, n (%)				< 0.001		< 0.001
Clear-cell	20,978 (75.3%)	426 (64.0%)	12,538 (81.7%)		8,014 (67.6%)	
Papillary	2,942 (10.6%)	95 (14.3%)	996 (6.5%)		1,851 (15.6%)	
Chromophobe	2,383 (8.6%)	30 (4.5%)	843 (5.5%)		1,510 (12.7%)	
Variant histology ^c^	1,556 (5.6%)	115 (17.3%)	966 (6.3%)		475 (4.0%)	
High-grade tumor ^d^, n (%)	9,273 (55.6%)	202 (74.0%)	5,918 (60.9%)	< 0.001	3,153 (47.1%)	< 0.001
Lymph node invasion ^e^, n (%)	4,785 (17.2%)	325 (48.8%)	3,009 (19.6%)	< 0.001	1,451 (12.2%)	< 0.001

^a^ Wilcoxon rank-sum test for continuous variables; Pearson’s chi-squared test for categorical variables; Ref.: pT4

^b^ annual income below median (80,000 USD)

^c^ including collecting duct carcinoma, sarcomatoid dedifferentiation, medullary carcinoma, hereditary leiomyomatosis and renal cell cancer-associated tumors, mesenchymal tumors, mucinous tumors, neuroendocrine tumors, and rhabdoid tumors

^d^ Fuhrman tumor grade G3–4 (only applicable for clear-cell and papillary histological subtype)

^e^ only applicable when lymphadenectomy was performed

Patients with pT4 harbored the largest tumors (median: 10.5 cm), followed by pT2 (median 9.0 cm, *p* < 0.001) and pT3 (median 8.0 cm, *p* < 0.001) in that order. Histological subtype distribution also significantly differed in both stage comparisons (pT4 versus pT3 and pT4 versus pT2, both *p* < 0.001). Specifically, clear-cell carcinoma represented the predominant histological subtype across all stages (pT4: 64.0%, pT3: 81.7%, pT2: 67.6%) and was particularly pronounced in pT3. Papillary and chromophobe histological subtypes were most prevalent in pT2 (15.6% and 12.7%, respectively). Conversely, lower proportions were observed in pT4 (14.3% and 4.5%) and pT3 (6.5% and 5.5%). Variant histology was substantially more frequent in pT4 (17.3%) compared with pT3 (6.3%) or pT2 (4.0%). Moreover, pT4 patients harbored the highest proportion of high-grade tumors (74.0%), followed by pT3 (60.9%, *p* < 0.001) and pT2 (47.1%, *p* < 0.001) in that order. Lymph node invasion was observed in 48.8% of pT4 patients, significantly higher than in pT3 (19.6%, *p* < 0.001) and pT2 (12.2%, *p* < 0.001) patients. Marginal, albeit statistically significant differences existed in year of diagnosis (median: 2012 vs. 2014 vs. 2015) and age (median: 62 vs. 64 vs. 61 years, all *p* ≤ 0.002).

### Primary analysis

After one-to-one PSM for all available patient and tumor characteristics, all 666 pT4 patients, 666 of 15,343 (4.3%) pT3 patients, and 666 of 11,850 (5.6%) pT2 patients were included in subsequent cumulative incidence analyses and multivariable CRR models (Table 2).

**Table 2:** Descriptive characteristics of pT2–4 renal cell carcinoma patients treated with radical nephrectomy within the SEER database (2004–2022), after 1:1 propensity score matching of pT4 to pT3 and to pT2 relying on year of diagnosis, age, sex, race/ethnicity, marital status, income, area of residence, tumor size, histological subtype/grade, and lymph node invasion

Characteristics	pT4 *n* = 666 (100%)	pT3 *n* = 666 (4.3%)	*p*-value ^a, f^	pT2 *n* = 666 (5.6%)	*p*-value ^a, f^
Year of diagnosis, median (IQR)	2012 (2008, 2018)	2012 (2008, 2018)	> 0.9	2013 (2008, 2016)	0.8
Age (in years), median (IQR)	62 (55, 72)	63 (54, 72)	0.7	63 (55, 71)	0.6
Male sex, n (%)	431 (64.7%)	433 (65.0%)	> 0.9	414 (62.2%)	0.3
Non-Caucasian race/ethnicity, n (%)	234 (35.1%)	229 (34.4%)	0.8	232 (34.8%)	> 0.9
Married, n (%)	412 (61.9%)	422 (63.4%)	0.6	411 (61.7%)	> 0.9
Low income ^b^, n (%)	356 (53.5%)	356 (53.5%)	> 0.9	348 (52.3%)	0.7
Rural residence, n (%)	90 (13.5%)	85 (12.8%)	0.7	96 (14.4%)	0.6
Tumor size (in cm), median (IQR)	10.5 (8.0, 13.2)	10.0 (7.5, 13.0)	0.1	10.0 (8.0, 12.0)	0.1
Histological subtype, n (%)			0.8		0.5
Clear-cell	426 (64.0%)	420 (63.1%)		449 (67.4%)	
Papillary	95 (14.3%)	90 (13.5%)		88 (13.2%)	
Chromophobe	30 (4.5%)	27 (4.1%)		22 (3.3%)	
Variant histology ^c^	115 (17.3%)	129 (19.4%)		107 (16.1%)	
High-grade tumor ^d^, n (%)	202 (74.0%)	194 (72.9%)	0.9	207 (71.4%)	0.5
Lymph node invasion ^e^, n (%)	325 (48.8%)	323 (48.5%)	0.6	327 (49.1%)	> 0.9

^a^ Wilcoxon rank-sum test for continuous variables; Pearson’s chi-squared test for categorical variables; Ref.: pT4

^b^ annual income below median (80,000 USD)

^c^ including collecting duct carcinoma, sarcomatoid dedifferentiation, medullary carcinoma, hereditary leiomyomatosis and renal cell cancer-associated tumors, mesenchymal tumors, mucinous tumors, neuroendocrine tumors, and rhabdoid tumors

^d^ Fuhrman tumor grade G3–4 (only applicable for clear-cell and papillary histological subtype)

^e^ only applicable when lymphadenectomy was performed

^f^ after PSM all covariates were well balanced with standardized mean differences < 0.1

**Fig. 1:**
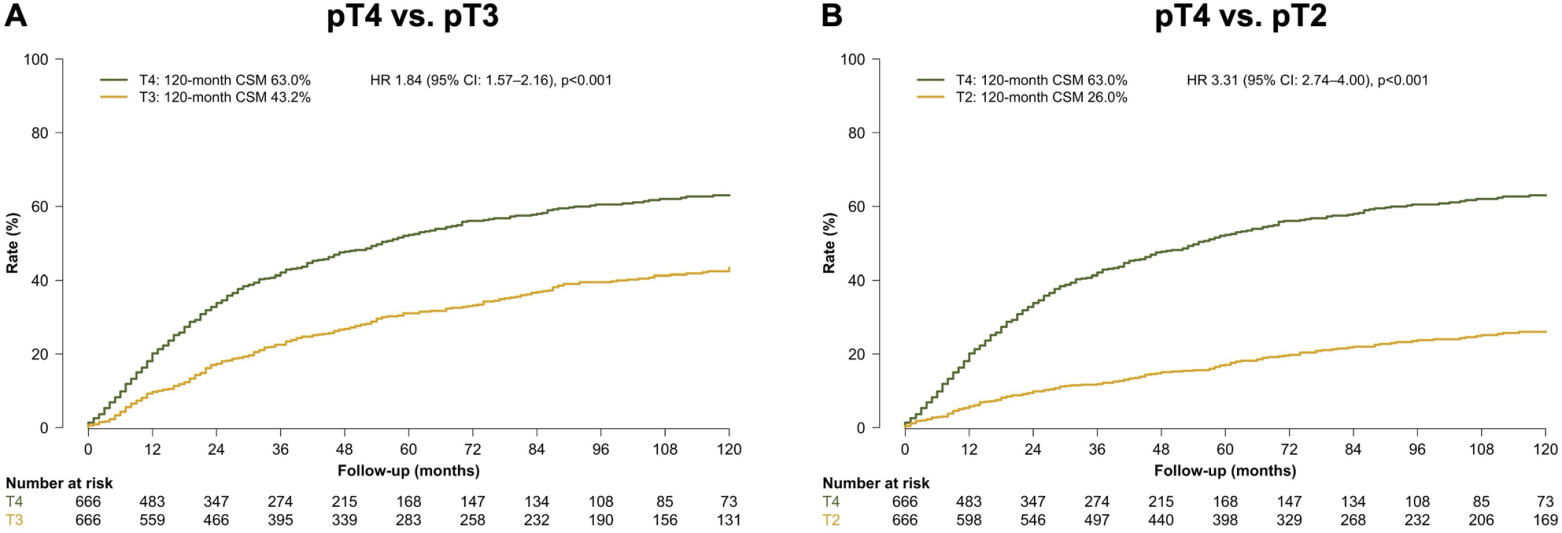
Cumulative incidence plots depicting cancer-specific mortality (CSM) of renal cell carcinoma patients treated with radical nephrectomy within the SEER database (2004–2022), stratified according to pT4 versus pT3 (**A**) and versus pT2 (**B**), after 1:1 propensity score matching. Hazard ratios (HR) derived from multivariable competing risks regression models adjusted for year of diagnosis, age, sex, race/ethnicity, marital status, income, area of residence, tumor size, histological subtype/grade, and lymph node invasion

At 120 months after nephrectomy, CSM rates were 63.0% in patients with pT4 compared with 43.2% in those with pT3 (Fig. 1A) and 26.0% in those with pT2 (Fig. 1B). In multivariable CRR models, pT4 stage was associated with a 1.84-fold higher risk of CSM relative to pT3 (95% confidence interval [CI]: 1.57–2.16, *p* < 0.001) and a 3.31-fold higher risk of CSM relative to pT2 (95% CI: 2.74–4.00, *p* < 0.001).

### Sensitivity analyses

#### Patients with clear-cell RCC

In the sensitivity analysis restricted to patients with clear-cell RCC, separate one-to-one PSM was applied. After PSM, all 426 pT4 patients, 426 of 12,538 (3.4%) pT3 patients, and 426 of 8,014 (5.3%) pT2 patients were included in cumulative incidence analyses and multivariable CRR models (Supplementary Table 1). At 120 months after nephrectomy, CSM rates were 61.6% in patients with pT4 compared with 40.2% in those with pT3 and 29.6% in those with pT2 (Supplementary Fig. 1). In multivariable CRR models, pT4 remained associated with a 1.93-fold higher risk of CSM relative to pT3 (95% CI: 1.56–2.38, *p* < 0.001) and a 2.96-fold higher risk of CSM relative to pT2 (95% CI: 2.36–3.71, *p* < 0.001).

#### Patients diagnosed before adoption of adjuvant systemic therapy

In the sensitivity analysis restricted to patients diagnosed before approval of adjuvant systemic therapy, separate one-to-one PSM was applied. After PSM, all 472 pT4 patients, 472 of 8,772 (5.4%) pT3 patients, and 472 of 9,000 (5.2%) pT2 patients were included in cumulative incidence analyses and multivariable CRR models (Supplementary Table 2). At 120 months after nephrectomy, CSM rates were 65.5% in patients with pT4 compared with 41.1% in those with pT3 and 26.1% in those with pT2 (Supplementary Fig. 2). In multivariable CRR models, pT4 remained associated with a 1.87-fold higher risk of CSM relative to pT3 (HR 1.87, 95% CI: 1.56–2.24, *p* < 0.001) and a 3.47-fold higher risk of CSM relative to pT2 (HR 3.47, 95% CI: 2.83–4.27, *p* < 0.001).

#### Patients without lymph node invasion

In the sensitivity analysis restricted to patients without lymph node invasion, separate one-to-one PSM was applied. After PSM, all 341 pT4 patients, 341 of 12,334 (2.8%) pT3 patients, and 341 of 10,399 (3.3%) pT2 patients were included in cumulative incidence analyses and multivariable CRR models (Supplementary Table 3). At 120 months after nephrectomy, CSM rates were 54.2% in patients with pT4 compared with 35.9% in those with pT3 and 23.4% in those with pT2 (Supplementary Fig. 3). In multivariable CRR models, pT4 remained associated with a 1.79-fold higher risk of CSM relative to pT3 (HR 1.79, 95% CI: 1.37–2.34, *p* < 0.001) and a 3.01-fold higher risk of CSM relative to pT2 (HR 3.01, 95% CI: 2.29–3.97, *p* < 0.001).

## Discussion

It is generally accepted that pT4 stage is associated with worse survival than pT3 or pT2 stage. However, it remains unclear whether the detrimental effect of pT4 stage is driven by unfavorable tumor characteristics such as larger tumor size, higher tumor grade, and less favorable histological subtypes, or whether it is independently attributable to adjacent organ invasion. Specifically, it is unknown whether, and if so to what extent, presence of pT4 increases the risk of CSM relative to pT3 and pT2 when all other patient and tumor characteristics are held constant. We addressed this knowledge gap and made several noteworthy observations.

First, we relied on a large, contemporary, population-based cohort of M0 RCC radical nephrectomy patients (SEER 2004–2022, *n* = 27,859). Within this sample, pT4 was rare (2.4%). Despite its small proportion, the absolute number of individuals harboring pT4 M0 stage (*n* = 666) markedly exceeds the sample sizes reported in prior studies (*n* = 22–49) [8, 9, 14, 15]. The relative proportions of pT3 and pT2 tumors were comparable to those reported in contemporary radical nephrectomy cohorts, supporting the external validity of our study cohort [ 11–14].

Second, we focused on CSM in patients with pT4 M0 RCC. Studies that specifically addressed this patient subgroup have not been reported. However, several investigators included pT4 in multivariable analyses evaluating the prognostic performance of TNM staging when survival represented the endpoint [8–15]. Most relied on CSM as was done in the current study. However, these previous studies examined the effect of pT4 stage along with all other T stages within heterogeneous cohorts including patients harboring distant metastasis (M1). Consequently, the incremental prognostic impact of pT4 within the M0 setting has remained insufficiently defined. To address this limitation, our analysis focused exclusively on M0 patients undergoing radical nephrectomy and performed stage-specific comparisons of pT4 versus pT3 and pT4 versus pT2, thereby ensuring clinically meaningful comparator groups.

Third, we addressed differences in tumor and patient characteristics between pT4, pT3 and pT2 patients. In those analyses, pT4 patients distinguished themselves based on larger tumor size, substantially higher proportion of variant histology, higher rates of high-grade tumors, and more frequent lymph node invasion. Based on these marked imbalances, it could be postulated that the more aggressive phenotype observed in pT4 tumors partially or entirely originates from adverse clinicopathologic features, but not from adjacent organ invasion (pT4) per se. Alternatively, it could be postulated that, after holding all these characteristics constant in comparisons of pT4 versus pT3 and pT4 versus pT2 tumors, patients harboring pT4 stage may still exhibit higher CSM. To isolate the independent prognostic effect of pT4 beyond differences in tumor size, other pathological characteristics, and demographic factors, we relied on the strictest statistical methodology, namely PSM followed by multivariable CRR models. To the best of our knowledge, no previous study controlled for tumor and patient characteristics with the attempt to test whether, and if so to what extent, pT4 confers an independent increase in CSM relative to pT3 and to pT2.

Fourth, CSM rates after 120 months were 63.0% in pT4 versus 43.2% in pT3, after holding all tumor and patient characteristics constant using most detailed PSM. In multivariable CRR models, pT4 was independently associated with a 1.8-fold higher CSM relative to pT3. In the separate comparison of pT4 versus pT2, 120-month CSM rates were 63.0% versus 26.0%, respectively, and pT4 remained associated with a 3.3-fold higher CSM after adjustment. These observations indicate that the presence of pT4 confers an independent increase in CSM, that is highly significant relative to both pT3 and pT2 patients. Notably, the magnitude of this effect was greater in the comparison with pT2 than with pT3 tumors.

Fifth, we performed several sensitivity analyses to evaluate the robustness of our findings. First, analyses were repeated in patients with clear-cell RCC only, given the predominance of this histological subtype and the potential biological heterogeneity between RCC subtypes. Second, analyses were repeated in patients diagnosed before the widespread adoption of adjuvant systemic therapy to address the possibility of differential treatment exposure. Third, analyses were repeated after exclusion of patients with lymph node invasion to determine whether the observed prognostic effect of pT4 persisted independently of regional lymphatic metastasis. Across all sensitivity analyses, effect estimates remained highly consistent with the primary analyses. These observations further strengthen the internal validity of the observed prognostic impact of pT4 stage and indicate that the association is unlikely to be primarily driven by histological subtype, differential use of adjuvant systemic therapy, or the presence of lymph node invasion.

Taken together, our findings indicate that the CSM disadvantage observed in pT4 M0 patients cannot be fully explained by accompanying adverse patient or tumor characteristics but rather appears at least partially attributable to adjacent organ invasion itself. Moreover, the clear stepwise increase in CSM from pT2 to pT3 to pT4 supports the current T-staging and underscores the prognostic relevance of adjacent organ invasion as the defining feature of pT4 stage.

The clinical implications of these observations are twofold. First, the markedly increased CSM observed in pT4 patients despite adjustment for other adverse clinicopathologic characteristics suggests that this subgroup warrants particular attention during postoperative risk stratification and follow-up planning. Second, the observed adverse prognosis identifies pT4 patients as a particularly high-risk population that could be prioritized for discussions regarding adjuvant systemic therapy. However, these implications should be regarded as hypothesis-generating, since the current study did not directly evaluate surveillance strategies or treatment efficacy and therefore cannot define optimal follow-up schedules or quantify the benefit of adjuvant treatment in this population. Importantly, current evidence and guideline recommendations for adjuvant immunotherapy primarily apply to patients with clear-cell RCC [21–24]. Consequently, only 64% of pT4 patients in our cohort would currently be considered eligible for such treatment strategies, whereas evidence for non-clear-cell RCC remains limited and further investigation is warranted.

Several limitations inherent to retrospective, registry-based analyses should be acknowledged. First, SEER provides detailed pathological staging information, but lacks data on preoperative imaging findings, intraoperative assessment, and surgeon-reported suspicion of adjacent organ invasion. As a result, discrepancies between clinical (cT4) and pathological (pT4) staging could not be directly evaluated, although the predictive accuracy of cT4 for true pT4 is known to be limited [2–9]. Second, information on surgical complexity, the specific adjacent organ involved, and the extent of its resection is not captured. Consequently, we could not account for the potential heterogeneity of pT4 disease according to the invaded structure. Third, pathological assessment is not centrally reviewed, and misclassification of pathological stage cannot be entirely excluded. Fourth, although tumor size and stage are reliably recorded, other potentially relevant pathological features, such as surgical margin status, lymphovascular invasion, tumor necrosis, or papillary RCC subtype (type 1 vs. type 2) are not available in SEER. Positive surgical margins have previously been associated with adverse oncologic outcomes after nephrectomy [25, 26]. In addition, information on individual patient comorbidities and performance status as well as use of adjuvant systemic therapy is lacking. Consequently, despite the use of PSM and multivariable adjustment, residual confounding from unmeasured variables remains possible. However, OCM was treated as a competing risk event and may, to some extent, capture the burden of relevant comorbid conditions. Furthermore, because pT4 patients are more likely to receive adjuvant systemic therapy, differential treatment exposure may have influenced the observed outcomes. Nevertheless, effect estimates remained highly consistent in a sensitivity analysis restricted to patients diagnosed before the widespread adoption of adjuvant systemic therapy. Fifth, the current study was restricted to surgically treated patients with pathologically confirmed disease [27, 28]. Consequently, patients with unresectable or clinically inoperable cT4 tumors were not captured, potentially introducing selection bias and limiting generalizability to all patients with locally advanced RCC. Furthermore, a substantial number of patients were excluded because of missing information regarding baseline characteristics or follow-up. Consequently, such complete-case analysis approach was required, which may have introduced selection bias if excluded patients systematically differed from those included in the final study cohort. Finally, SEER does not report recurrence-related endpoints, such as disease-free, progression-free, or metastasis-free survival, which restricted our evaluation to CSM.

## Supplementary Information

Supplementary Material 1.

## Data Availability

The used data are derived from the SEER database of the National Cancer Institute. Access to SEER data is available upon request through the SEER*Stat software after registration and approval by the National Cancer Institute. The authors had authorized access to these data. The data are not publicly available due to privacy and confidentiality restrictions imposed by the SEER program.
