# Supplementary material for: Prognostic impact of adjacent organ invasion on cancer-specific mortality in non-metastatic renal cell carcinoma
Source: World J Surg Oncol. 2026 Jul 20;24:304. doi: 10.1186/s12957-026-04511-3 (PMC13383379; doi:10.1186/s12957-026-04511-3)

Supplemental Table 1. Descriptive characteristics of pT2–4 clear-cell renal cell carcinoma patients treated with radical nephrectomy within the SEER database (2004–2022), after 1:1 propensity score matching of pT4 to pT3 and to pT2 relying on year of diagnosis, age, sex, race/ethnicity, marital status, income, area of residence, tumor size, tumor grade, and lymph node invasion.

**Characteristics**	**pT4 n = 426 (100%)**	**pT3 n = 426 (3.4%)**	**p-value** [a,e]	**pT2 n = 426 (5.3%)**	**p-value** [a,e]
**Year of diagnosis**, median (IQR)	2011 (2008, 2018)	2011 (2007, 2015)	0.3	2012 (2008, 2016)	0.8
**Age** (in years), median (IQR)	62 (56, 72)	63 (55, 71)	0.8	63 (55, 72)	>0.9
**Male sex**, n (%)	276 (64.8%)	277 (65.0%)	>0.9	278 (65.3%)	0.9
**Non-Caucasian race/ethnicity**, n (%)	135 (31.7%)	127 (29.8%)	0.6	137 (32.2%)	0.9
**Married**, n (%)	264 (62.0%)	255 (59.9%)	0.5	278 (65.3%)	0.3
**Low income** [b], n (%)	229 (53.8%)	215 (50.5%)	0.3	227 (53.3%)	0.9
**Rural residence**, n (%)	52 (12.2%)	41 (9.6%)	0.2	59 (13.8%)	0.5
**Tumor size** (in cm), median (IQR)	10.5 (8.2, 13.0)	10.2 (7.5, 12.7)	0.1	9.8 (8.3, 12.0)	0.1
**High-grade tumor** [c], n (%)	183 (74.7%)	173 (73.9%)	0.7	172 (69.9%)	0.5
**Lymph node invasion** [d], n (%)	189 (81.8%)	211 (86.5%)	0.3	197 (82.4%)	0.8

[a] Wilcoxon rank-sum test for continuous variables; Pearson’s chi-squared test for categorical variables; Ref.: pT4

[b] annual income below median (80,000 USD)

[c] Fuhrman tumor grade G3–4

[d] only applicable when lymphadenectomy was performed

[e] after PSM all covariates were well balanced with standardized mean differences <0.1

Supplemental Figure 1. Cumulative incidence plot depicting cancer-specific mortality (CSM) of clear-cell renal cell carcinoma patients treated with radical nephrectomy within the SEER database (2004–2022), stratified according to pT4 versus pT3 (A) and versus pT2 (B), after 1:1 propensity score matching. Hazard ratios (HR) derived from multivariable competing risks regression models adjusted for year of diagnosis, age, sex, race/ethnicity, marital status, income, residence, tumor size, tumor grade, and lymph node invasion.

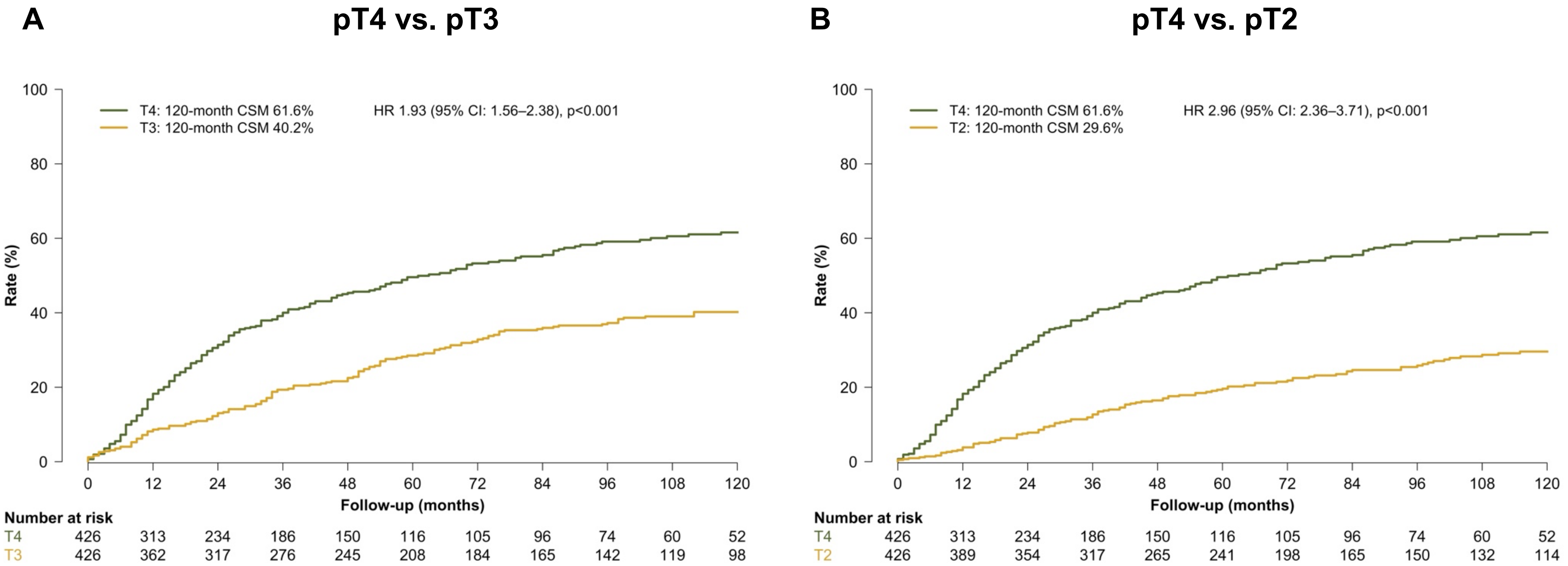

Supplemental Table 2. Descriptive characteristics of pT2–4 renal cell carcinoma patients treated with radical nephrectomy within the SEER database (2004–2017), after 1:1 propensity score matching of pT4 to pT3 and to pT2 relying on year of diagnosis, age, sex, race/ethnicity, marital status, income, area of residence, tumor size, histological subtype/grade, and lymph node invasion.

Characteristics	pT4<br>n = 472<br>(100%)	pT3<br>n = 472<br>(5.4%)	p-value [a,f]	pT2<br>n = 472<br>(5.2%)	p-value [a,f]
**Year of diagnosis**, median (IQR)	2009 (2007, 2012)	2010 (2007, 2012)	0.3	2009 (2006, 2012)	0.3
**Age** (in years), median (IQR)	61 (55, 71)	63 (54, 71)	0.7	62 (53, 70)	0.5
**Male sex**, n (%)	316 (66.9%)	310 (65.7%)	0.7	308 (65.3%)	0.6
**Non-Caucasian race/ethnicity**, n (%)	152 (32.2%)	143 (30.3%)	0.5	166 (35.2%)	0.3
**Married**, n (%)	301 (63.8%)	295 (62.5%)	0.7	306 (64.8%)	0.7
**Low income** [b], n (%)	269 (57.0%)	276 (58.5%)	0.6	284 (60.2%)	0.3
**Rural residence**, n (%)	56 (11.9%)	60 (12.7%)	0.7	59 (12.5%)	0.8
**Tumor size** (in cm), median (IQR)	10.0 (7.9, 13.0)	10.0 (7.5, 13.0)	0.7	9.5 (8.0, 11.5)	0.3
**Histological subtype**, n (%)			>0.9		0.2
Clear-cell	312 (66.1%)	317 (67.2%)		336 (71.2%)	
Papillary	80 (16.9%)	77 (16.3%)		79 (16.7%)	
Chromophobe	23 (4.9%)	23 (4.9%)		18 (3.8%)	
Variant histology [c]	57 (12.1%)	55 (11.7%)		39 (8.3%)	
**High-grade tumor** [d], n (%)	115 (76.2%)	106 (65.8%)	0.1	117 (72.7%)	0.5
**Lymph node invasion** [e], n (%)	289 (61.2%)	294 (62.3%)	0.7	300 (63.6%)	0.6

[a] Wilcoxon rank-sum test for continuous variables; Pearson's chi-squared test for categorical variables; Ref.: pT4

[b] annual income below median (80,000 USD)

[c] including collecting duct carcinoma, sarcomatoid dedifferentiation, medullary carcinoma, hereditary leiomyomatosis and renal cell cancer-associated tumors, mesenchymal tumors, mucinous tumors, neuroendocrine tumors, and rhabdoid tumors

[d] Fuhrman tumor grade G3–4 (only applicable for clear-cell and papillary histological subtype)

[e] only applicable when lymphadenectomy was performed

[f] after PSM all covariates were well balanced with standardized mean differences <0.1

Supplemental Figure 2. Cumulative incidence plot depicting cancer-specific mortality (CSM) of cell carcinoma patients treated with radical nephrectomy within the SEER database (2004–2017), stratified according to pT4 versus pT3 (A) and versus pT2 (B), after 1:1 propensity score matching. Hazard ratios (HR) derived from multivariable competing risks regression models adjusted for year of diagnosis, age, sex, race/ethnicity, marital status, income, area of residence, tumor size, histological subtype/grade, and lymph node invasion.

A **pT4 vs. pT3** **B** **pT4 vs. pT2**

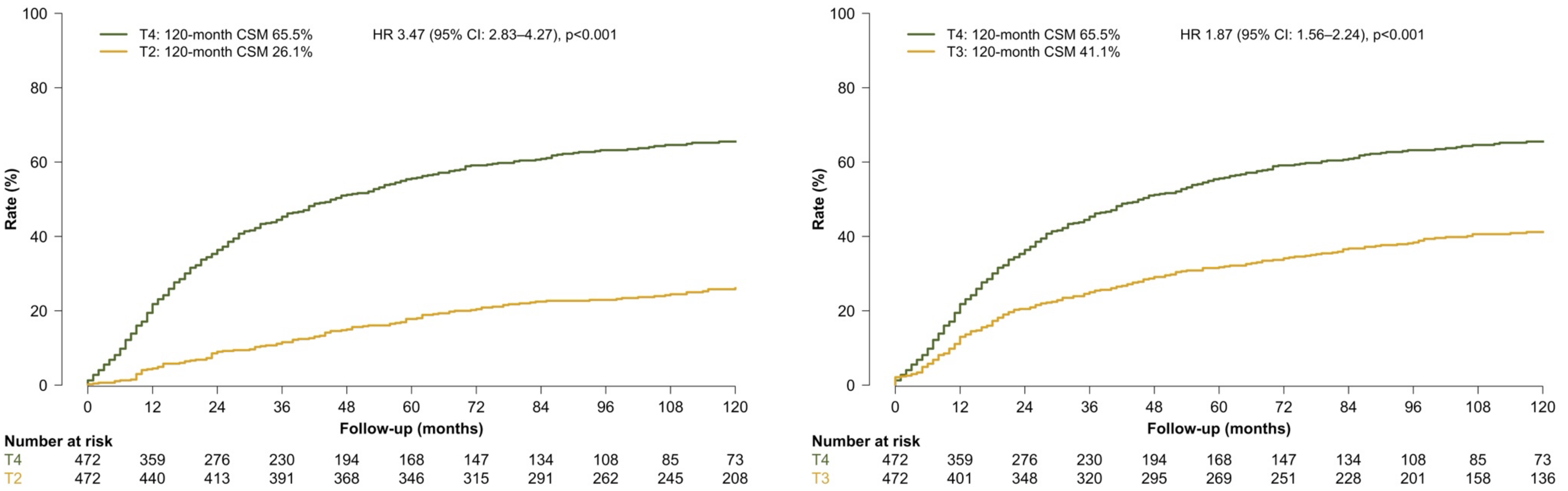

Supplemental Table 3. Descriptive characteristics of pT2–4 renal cell carcinoma patients without lymph node invasion treated with radical nephrectomy within the SEER database (2004–2022), after 1:1 propensity score matching of pT4 to pT3 and to pT2 relying on year of diagnosis, age, sex, race/ethnicity, marital status, income, area of residence, tumor size, and histological subtype/grade.

**Characteristics**	**pT4 n = 341 (100%)**	**pT3 n = 341 (2.8%)**	**p-value** [a,e]	**pT2 n = 341 (3.3%)**	**p-value** [a,e]
**Year of diagnosis**, median (IQR)	2015 (2010, 2020)	2015 (2010, 2020)	0.9	2016 (2011, 2018)	0.3
**Age** (in years), median (IQR)	64 (57, 73)	63 (57, 70)	0.4	66 (56, 75)	0.7
**Male sex**, n (%)	213 (62.5%)	212 (62.2%)	>0.9	220 (64.5%)	0.6
**Non-Caucasian race/ethnicity**, n (%)	125 (36.7%)	115 (33.7%)	0.4	118 (34.6%)	0.6
**Married**, n (%)	198 (58.1%)	204 (59.8%)	0.6	195 (57.2%)	0.8
**Low income** [b], n (%)	185 (54.3%)	191 (56.0%)	0.6	188 (55.1%)	0.8
**Rural residence**, n (%)	48 (14.1%)	53 (15.5%)	0.6	54 (15.8%)	0.5
**Tumor size** (in cm), median (IQR)	10.0 (7.8, 13.0)	10.0 (7.3, 12.7)	0.5	9.5 (8.0, 12.0)	0.4
**Histological subtype**, n (%)			0.8		>0.9
Clear-cell	237 (69.5%)	242 (71.0%)		230 (67.4%)	
Papillary	28 (8.2%)	25 (7.3%)		32 (9.4%)	
Chromophobe	13 (3.8%)	9 (2.6%)		14 (4.1%)	
Variant histology [c]	63 (18.5%)	65 (19.1%)		65 (19.1%)	
**High-grade tumor** [d], n (%)	126 (68.9%)	124 (69.7%)	>0.9	122 (69.7%)	0.8

[a] Wilcoxon rank-sum test for continuous variables; Pearson's chi-squared test for categorical variables; Ref.: pT4

[b] annual income below median (80,000 USD)

[c] including collecting duct carcinoma, sarcomatoid dedifferentiation, medullary carcinoma, hereditary leiomyomatosis and renal cell cancer-associated tumors, mesenchymal tumors, mucinous tumors, neuroendocrine tumors, and rhabdoid tumors

[d] Fuhrman tumor grade G3–4 (only applicable for clear-cell and papillary histological subtype)

[e] after PSM all covariates were well balanced with standardized mean differences <0.1

Supplemental Figure 3. Cumulative incidence plot depicting cancer-specific mortality (CSM) of cell carcinoma patients without lymph node invasion treated with radical nephrectomy within the SEER database (2004–2022), stratified according to pT4 versus pT3 (A) and versus pT2 (B), after 1:1 propensity score matching. Hazard ratios (HR) derived from multivariable competing risks regression models adjusted for year of diagnosis, age, sex, race/ethnicity, marital status, income, area of residence, tumor size, and histological subtype/grade.

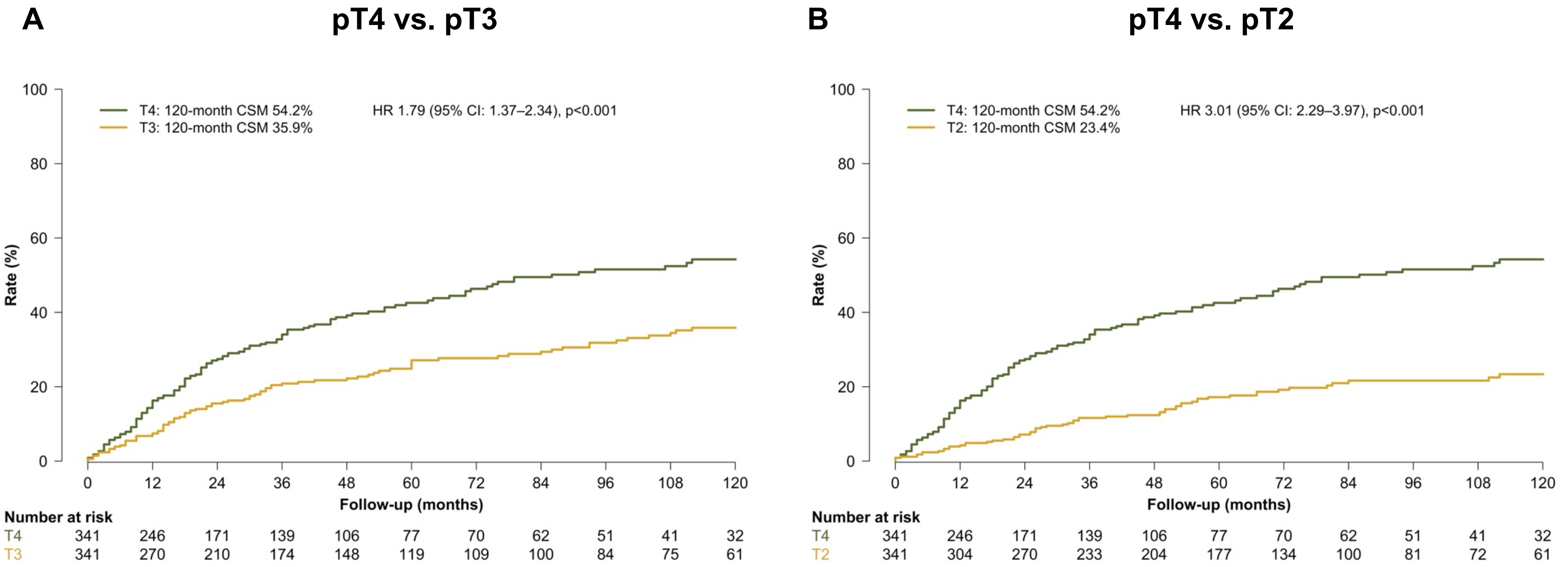

Supplement: Supplementary file 1 — Supplementary Material 1. [file 12957_2026_4511_MOESM1_ESM.pdf]
